# Effectiveness of Keishibukuryogan on Chronic-Stage Lichenification Associated with Atopic Dermatitis

**DOI:** 10.5402/2012/158598

**Published:** 2012-11-14

**Authors:** Megumi Mizawa, Teruhiko Makino, Hiroaki Hikiami, Yutaka Shimada, Tadamichi Shimizu

**Affiliations:** ^1^Department of Dermatology, Graduate School of Medicine and Pharmaceutical Sciences, University of Toyama, Sugitani, Toyama 2630, Japan; ^2^Department of Japanese Oriental Medicine, Graduate School of Medicine and Pharmaceutical Sciences, University of Toyama, Sugitani, Toyama 2630, Japan

## Abstract

Atopic dermatitis (AD) is a common inflammatory skin disease with recurring episodes of itching and a chronic relapsing course. Keishibukuryogan (KBG) is a traditional herbal medicine, composed of five kinds of medical plants and has been administered to patients with blood stagnation in Japan. This study investigated the effect of KBG on the disease activity in AD (*n* = 45) patients. AD patients were administered KBG for 4 to 6 weeks in addition to their prescribed medications. The results showed that the SCORAD index and VAS score were significantly decreased after the administration of KBG (*P* < 0.01). KBG also decreased the serum LDH level significantly (*P* < 0.01). The global assessment of the clinical response in SCORAD index showed that 88.5% of the patients with moderate improvement to excellent response (*n* = 26) had a high lichenification score (lichenification score ≥2 in SCORAD). On the other hand, only 42.1% of the patients with no improvement to mild improvement (*n* = 19) had a high lichenification score. Furthermore, long-term administration of KBG for 9–67 weeks showed a marked improvement in patients with a high lichenification score. Therefore, KBG was found to be effective against AD, particularly in cases presenting with lichenified lesions.

## 1. Introduction

Atopic dermatitis (AD) is a common chronic inflammatory skin disease characterized by inflammatory infiltration, extensive pruritus, and a clinical course of symptomatic flares and remissions. The pathogenesis of the disease is now better understood, and important factors involved in the pathogenesis of this disease are genetic factors, skin barrier dysfunction, and immune dysregulation [1]. Lesions are often erythematous with edematous, weeping papules, and vesicles in the acute AD and lichenified thickened plaques in the chronic stage. The current treatment options for AD include topical agents, such as topical corticosteroids and oral antiallergic drugs. Effective long-term treatment is sometimes difficult due to the chronic, relapsing nature of this disease, thus creating a need to find better therapeutic options with minimal side effects that are well tolerated over the variable course of this disease.

Traditional herbal medicine has a long history, and it contributes to the prevention and treatment of various diseases. To date, only a few reports regarding the efficacy of traditional herbal medicines as a treatment option for AD have been reported [2–5]. Recent double-blind, placebo-controlled study showed considerably effective benefits in managing clinical signs of AD with Hochu-ekki-to [6]. 

Keishibukuryogan (KBG, Gui-zhi-fu-ling-wan in Chinese) is a traditional herbal medicine that has been widely administered to patients with blood stagnation for improving blood circulation. Matsumoto et al. explored a proteomic approach for the diagnosis of blood stasis in rheumatoid arthritis patients treated with KBG [7]. In addition, KBG is used to treat symptoms of peripheral ischemia such as cold extremities [8]. KBG is now one of the most frequently used traditional medicines in Japan and has been used clinically to treat various diseases, including skin diseases. 

The purpose of this study was to evaluate the clinical effect of KBG on disease activity in AD patients in addition to conventional modalities.

## 2. Materials and Methods

### 2.1. Subjects

The study subjects included forty-five AD patients (23 males and 22 females; age, 14–41 years; mean age, 27.4 years). The diagnosis of AD was based on the Hanifin and Rajka criteria [9], and the patients had no other concomitant diseases.

### 2.2. Design of Study

The study was performed as a prospective self-controlled trial. These AD patients were administered KBG (TJ-25; 7.5 g per day) before meals three times a day for 4 to 6 weeks in addition to their prescribed medications, such as topical corticosteroids and oral antiallergic drugs. Patients were not allowed to take other medications during the study. Clinical and laboratory assessments were performed at week 0 and week 4 or 6. The study design was approved by the Human Subjects Committee, University of Toyama. All patients provided written informed consent in accordance with the ethical guidelines set forth in the 1975 Declaration of Helsinki.

### 2.3. Clinical Severity and Laboratory Assessments

AD patients were evaluated for general disease severity using the Scoring AD (SCORAD) index. The intensity part of the SCORAD index consists of six items: erythema, edema/papulation, excoriations, lichenification, oozing/crusts, and dryness. Each item can be graded on a scale 0–3. The subjective items include daily pruritus and sleeplessness. Both subjective items can be graded on a 10 cm visual analogue scale. The maximum subjective score is 20. A visual analogue scale (VAS) score (range 0–100) was used for the assessment of itching. A recently published study introduced a new evaluation tool, the global assessment of the clinical response for the SCORAD index, assigning a score of 0–4. Score 0–4 was defined as proportion of decrease in SCORAD values: 0—no improvement (0%); 1—mild improvement (1%–25%); 2—moderate improvement (26%–50%); 3—marked improvement (51%–75%); 4—excellent response (76%–100%) [10]. The serum lactate dehydrogenase (LDH) level, serum total IgE level, and peripheral blood eosinophils count were measured by laboratory tests.

### 2.4. Statistical Analysis

Data are presented as the mean values plus the standard error of the mean ± SE The statistical analysis was performed using paired *t*-test and the Wilcoxon signed-ranks test. A *P* value of less than 0.05 was considered to be statistically significant.

## 3. Results

### 3.1. Assessment of SCORAD Index and VAS Score in AD Patients Treated with KBG

**Figure 1:**
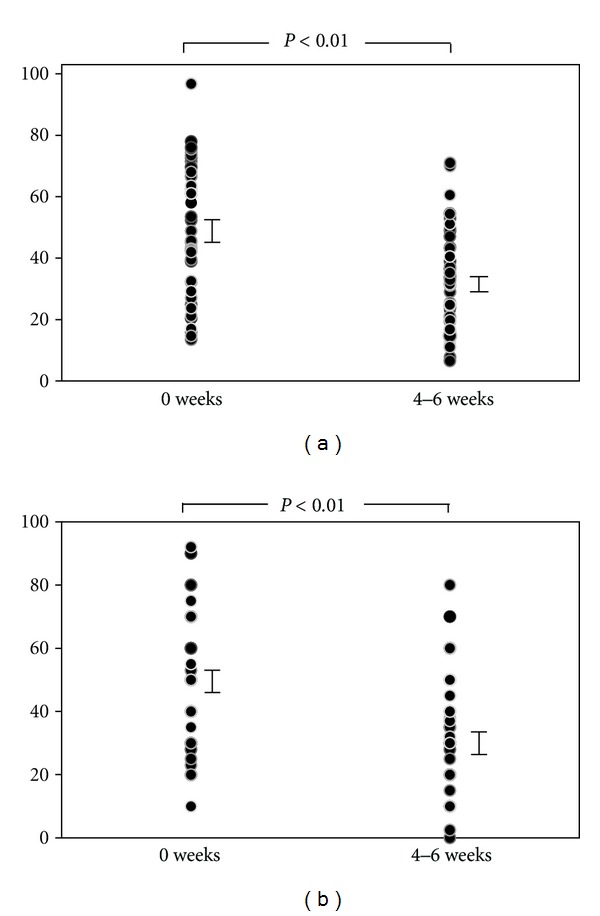
SCORAD index and VAS score in AD patients. (a) SCORAD index and (b) VAS score in patients with AD before and after the treatment with KBG. These index and scores were decreased significantly (*P* < 0.01).

**Figure 2:**
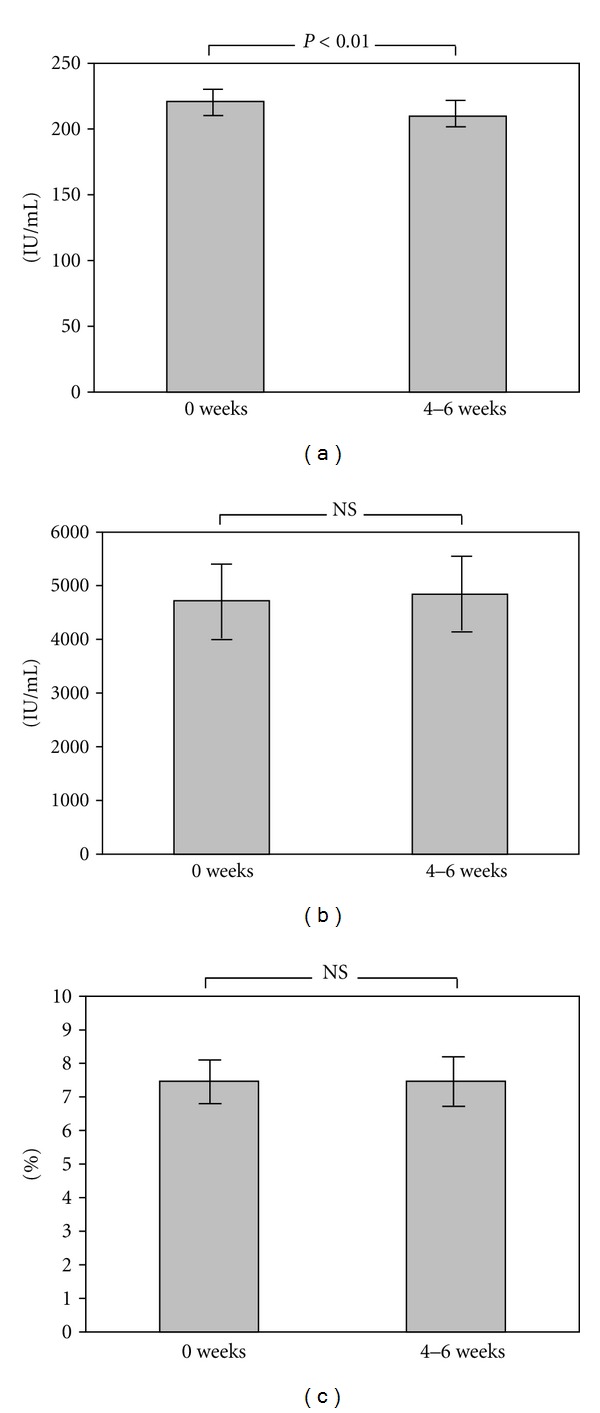
Serum level of LDH, IgE, and blood eosinophils. (a) The serum LDH level, (b) serum IgE level, (c) blood eosinophils in patients with AD before and after the treatment with KBG. The serum LDH level was decreased significantly (*P* < 0.01). However, no statistically significant differences were observed in IgE and eosinophils.

The results showed that the SCORAD index and VAS score were 49.84 ± 3.09 and 49.02 ± 3.08 before treatment with KBG. The SCORAD index and VAS score were decreased after the treatment with KBG (SCORAD index 49.84 ± 3.09 versus 33.32 ± 2.31, *P* < 0.01; VAS score 49.02 ± 3.08 versus 31.61 ± 3.02, *P* < 0.01; Figure 1). Therefore, treatment with KBG significantly improved AD-related objective and subjective symptoms. The serum LDH level was also significantly decreased (220.9 ± 10.8 versus 209.7 ± 10.3, *P* < 0.01; Figure 2(a)). However, no statistically significant differences were observed in IgE level and eosinophils count before and after the treatment (IgE 4722.1 ± 848.0 versus 4842.2 ± 843.9, *P* = 0.46; eosinophils count 7.47 ± 0.84 versus 7.47 ± 0.90, *P* = 0.74; Figures 2(b) and 2(c)).

### 3.2. The Global Assessment of the Clinical Response in SCORAD

**Figure 3:**
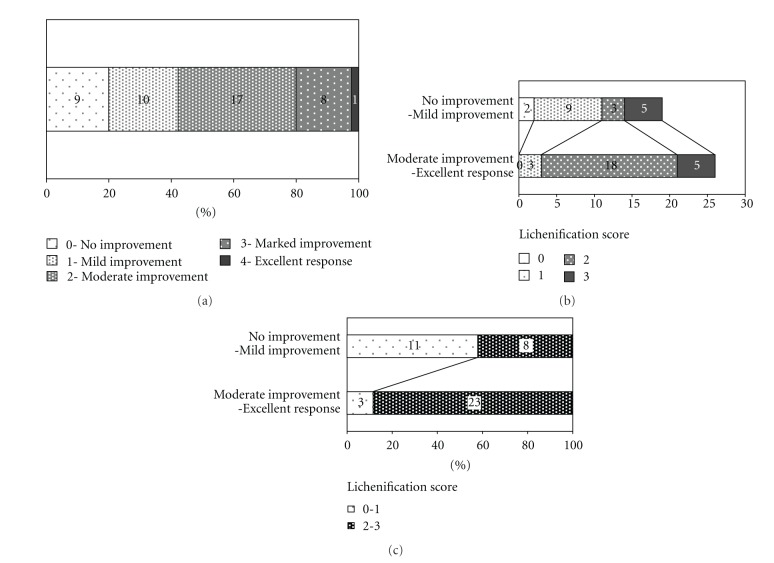
The global assessment of the clinical response in SCORAD index. (a) Details of the global assessment of the clinical response in SCORAD index among 45 AD patients. (b) Details of the lichenification score among the group of no improvement to mild improvement and the group of moderate improvement to excellent response patients. (c) Proportion of patient with high-to-low lichenification score among the group of no improvement to mild improvement and the group of moderate improvement to excellent response patients.

The global assessment of the clinical response in SCORAD index showed no improvement in 9 patients, mild improvement in 10 patients, moderate improvement in 17 patients, marked improvement in 8 patients, and an excellent response in 1 patient. The percentage of no improvement to mild improvement (0%–25%) was 42.2% and moderate improvement to excellent response (26%–100%) was 57.8% (Figure 3(a)). The group with moderate improvement to excellent response patients had a high lichenification score (88.5%, lichenification score ≥2 in SCORAD index). On the other hand, only 42.1% had a high lichenification score in the groups with no to mild improvement patients treated with KGB (Figures 3(b) and 3(c)).

### 3.3. Observation of Long-Term Followup in Patients Treated with KBG

Five patients continued the treatment with KBG following the end of the clinical study. All these patients had a high lichenification score. These 5 cases included 1 case with an excellent response, 1 case with a marked improvement, and 3 cases with a moderate improvement at the end of the clinical study. After the subsequent long-term administration of KBG for 9–67 weeks, 3 cases demonstrated an excellent response and 2 cases showed a marked improvement (Table 1).

### 3.4. Safety

No adverse reactions in laboratory data were noted in any patients. No adverse events, including those of unclear causality with treatment with KBG, were observed in the study.

## 4. Discussion

There has been increased interest in the use of traditional herbal medicine to develop new therapeutic agents without a corticosteroid for AD treatment. KBG is frequently used in traditional Japanese and Chinese herbal medicine to treat several symptoms, including skin diseases. The preparation has demonstrated anti-inflammatory and free-radical scavenging effects. KBG is composed of five medicinal plants, *Cinnamomum cassia *Blume (Cinnamomi cortex), *Paeonia lactiflora *Pallas (Paeoniae Radix), *Paeonia suffruticosa *Andrews (Moutan cortex), *Prunus persica *Batsch (Persicae semen), and *Poria cocos* Wolf (Hoelen) (Table 2) [11]. Paeonol, one of the main components of Moutan Cortex, has an antithrombotic effect. Galloylglucose of Paeoniae Radix and polyphenol of Cinnamomi Cortex, reportedly have endothelium-dependent relaxative effects, as well as antioxidant effects [12]. Therefore, these effects were assumed to positively influence the vascular function, radical generation, and so on. Previously, KBG has been reported to improve AD-related objective and subjective symptoms. The serum Thymus and activation-regulated chemokine (TARC/CCL17) level was also decreased in accordance with the improvement of AD treated with KBG [13]. TARC is a member of the CC chemokine superfamily, produced by monocyte-derived dendritic cells, endothelial cells, and keratinocytes. TARC is a selective chemoattractant for cells expressing CC chemokine receptor4 (CCR4), such as Th2-type cells [14]. These results suggest that KBG may have an inhibitory effect on Th2-type chemokine production in addition to the traditional activity. KBG could probably be used more effectively to treat inflammatory disease involving Th2-type chemokines, such as AD.

KBG improves conjunctional microcirculation in patients with cerebrospinal vascular diseases [15], thus suggesting that it may have beneficial effects on hematological parameters such as blood viscosity and red blood cell deformability [16–18]. In addition, KBG has beneficial effect on endothelial function in patients with metabolic syndrome-related factors [19]. AD lesions are characterized by differences in the activation state of endothelial cells and the release of inflammatory mediators by and toward the vasculature [20]. Longstanding inflammatory skin due to itch-induced scratching causes cutaneous damage including endothelial cells manifested as lichenification. In this study, most of the patients with moderate improvement to excellent response had a high lichenification score (88.5%). On the other hand, only 42.1% of the patients with no improvement to mild improvement had a high lichenification score. These findings might be supportive that the vascular system is ultimately involved in clinical symptoms of AD, such as the chronic stage of lichenification.

KBG has a favorable effect on impaired glucose metabolism in type 2 diabetes by improving glucose intolerance, and it has been suggested that some of these effects are derived from the reduction of the TNF-*α* content in skeletal muscle [21]. Paeoniae Radix and Moutan Cortex contain many known active components which are common in both, including paeoniflorin, paeonol, oxypaeoniflorin, benzoylpaeoniflorin, and palbinone [22]. Paeoniflorin is a characteristic main principal bioactive component of Paeoniae Radix in KBG, which includes approximately 5.57% (w/w) paeoniflorin, and Moutan Cortex, which includes approximately 3.96% (w/w) paeoniflorin [23]. Paeoniflorin has many pharmacological effects, including anti-inflammatory and antiallergic effects [24]. KBG and paeoniflorin suppress the production of MIF, IL-6, IL-8, and TNF-*α* in Lipopolysaccharide-stimulated human dermal microvessel endothelial cells, which are the prominent cells in dermal skin [25]. Accordingly, KBG may have beneficial effects that result in the inhibition of inflammatory cytokines in HDMECs.

The long-term administration of KBG demonstrated marked improvement in patients with a high lichenification score. Consequently, it was believed that such long-term administration may be effective if patients show a tendency of remission in symptoms due to KBG administration for about a month. Therefore, KBG was found to be effective against AD, particularly in cases with lichenified lesions. These findings suggested that KBG may become a useful treatment for intractable AD in patients that have been treated with conventional modalities. 

## Figures and Tables

**Table 1:** Long-term followup in patients treated with KBG.

Case	Age	Sex	Lichenification score (0 week)	Period (week)	SCORAD index	VAS score	SCORAD improvement (%)	Global assessment of clinical response in SCORAD
1	36	Female	2	0	69.5	70	—	—
				4	36.5	30	47.5	2
				43	11.2	10	83.9	4
				67	3.7	0	94.7	4

2	27	Male	2	0	42.5	20	—	—
				6	6.5	10	84.7	4
				15	11	20	74.1	3

3	30	Male	3	0	76	60	—	—
				5	54.5	35	28.3	2
				9	32.5	40	57.2	3

4	38	Female	2	0	43	50	—	—
				4	19.8	38	54.0	3
				26	10	20	76.7	4

5	18	Male	2	0	61	55	—	—
				4	40.5	50	33.6	2
				19	6.1	20	90	4

**Table 2:** Components of Keishibukuryogan (KBG).

Japanese name	Scientific name	Botanical name	Ratio (g)
Keihi	Cinnamomi cortex	*Cinnamomum cassia* Blume	1
Syakuyaku	Paeoniae radix	*Paeonia lactiflora* Pallas	1
Tounin	Persicae semen	*Prunus persica* Batsch	1
Bukuryou	Hoelen	*Poria cocos* Wolf	1
Botanpi	Moutan cortex	*Paeonia suffruticosa* Andrews	1

## Nonstandard Abbreviations

AD: Atopic dermatitis
KBG: Keishibukuryogan
LDH: Lactate dehydrogenase
SCORAD: SCORingAD
TARC: Thymus and activation-regulated chemokine
VAS: Visual analogue scale.
